# Predictors of European League Against Rheumatism (EULAR) good response, DAS-28 remission and sustained responses to TNF-inhibitors in rheumatoid arthritis: a prospective study in refractory disease

**DOI:** 10.1186/s40064-015-0979-6

**Published:** 2015-05-01

**Authors:** Reem Hamdy A Mohammed, Faisal Farahat, Hanady H Kewan, Mohammed A Bukhari

**Affiliations:** Rheumatology and Clinical Immunology, Department of Rheumatology and Rehabilitation, School of Medicine, Cairo University Hospitals, Cairo, Egypt; Internal Medicine Department, Alhada Armed Forces Hospital, Taif, Kingdom of Saudi Arabia; Community Medicine, Munofia University, Munofia, Egypt; Research Department Al Hada Armed Forces Hospital, Taif, Kingdom of Saudi Arabia; Department of Radio-diagnosis, Faculty of Medicine, Mansoura University, Mansoura, Egypt; Alhada Armed Forces Hospital, Taif, Kingdom of Saudi Arabia

**Keywords:** EULAR good response, DAS-28 remission, Sustained responses, Refractory rheumatoid arthritis, Tumor necrosis factor inhibitors

## Abstract

The aim of this study was to survey factors related to EULAR good response, the DAS-28 definition of remission, ACR 50 response, sustained response to tumor necrosis factor inhibitors (TNF-I) therapy in biologic naïve patients with refractory rheumatoid arthritis. This was a single center observational clinical prospective 2 years’ study, EULAR response criteria, DAS 28, HAQ and radiographic changes were recorded. Eighty patients included (64 females and 16 males, mean age was 48.4 + −17.9 years, mean disease duration 7.3 + −5.9 years). At 6 months 70% achieved EULAR good response, 51.8% achieved DAS-28 remission. Good response/sustained responses inversely correlated with baseline DAS-28 and radiographic erosions P <0.05. EULAR good response/remission by 6 months, sustained response at 2 years positively correlated with the decline in RF titers (r = 0.33, P < 0.05 & r = 0.30, P < 0.03 respectively), negatively correlated with the baseline HAQ. Regression analysis identified higher serum hemoglobin concentration, lower baseline HAQ scores, and the absence of radiographic erosions as significant predictors of good as well as sustained responses after adjustment for potential covariates. Methotrexate was associated with favorable responses and remission at 6 months (ORs = 1.13, 1.30 respectively). The study concluded that a lower baseline DAS-28 and HAQ scores, the lack of radiographic erosions favored EULAR good response and were significant predictors of sustained response to TNF-I.

## Introduction

Rheumatoid Arthritis is a systemic autoimmune inflammatory disease of indefinite etiology. The disease specifically affects the synovial joints contributing to significant synovial inflammation, progressive joint destruction and deformity of the affected joints. RA affects approximately 1% of adults all over the world with the diagnosis usually established between the third and fifth decade of life. Women are 2 to 3 times more likely to be diagnosed than men. The disease might additionally contribute to extra-articular multi-organ inflammation with variable outcome. Improperly treated the disease might lead to significant morbidity and incapacitation. For decades the conventional synthetic DMARDs (csDMARDs) have been the corner stone for the management of RA with methotrexate being the anchor drug in most of the designed regimens. Multiple studies and real life reports have documented that even with the highest standards of care with the use of csDMARDs either alone or in combinations up to 60% of the affected cases with RA fail to achieve successful remission. Within the last decade the field of therapeutics have witnessed an evolution in the treatment armamentarium of rheumatoid arthritis with the lead of the Tumor necrosis factor inhibitors (TNF-I) as the first line biologic drugs (Burmester GR et al. 2012; Wolfe and Michaud 2010; Nozaki et al. 2013; Mohammed et al. 2014; Nam et al. 2010; Hetland et al. 2010; Gartlehner et al. 2006; Wolfe and Michaud 2010; Gaujoux-Viala et al. 2010).

The aim of the current study was to prospectively survey the factors related to achieving an EULAR good/remission using the DAS-28 disease activity score and investigate predictors of a sustained response in a population of biologic naïve patients with refractory rheumatoid arthritis who receive tumor necrosis factor inhibitors TNF-I.

## Methods

This is a single center observational clinical study in a cohort of biologic naïve Refractory RA patients initiating their first line biologic treatment of TNF-inhibitors (infliximab, adalimumab or etanercept each administered based on an agreement between the patient and the treating physician with special considerations). The study was conducted at the Rheumatology section/Internal Medicine Department, Alhada Armed Forces Hospital, Kingdom of Saudi Arabia throughout a period of two years October 2010- October 2012. TNF-Is were given as recommended by the manufacturers (Infliximab 3-5 mg/kg intravenous infusion, Adalimumab 40 mg subcutaneous every 2 weeks, etanercept 50 mg subcutaneous weekly). Authors used the STROBE guidelines for observational studies for construction of their research checklist. The study was approved by the organizational ethics committee and was conducted in accordance with World Medical Association Helsinki Declaration of ethics (Adopted by the 18th WMA General Assembly, Helsinki, Finland, June 1964). All the patients included gave informed consents prior to the initiation of TNF-I biologic therapy.

Inclusion criteria:

- All patients who satisfied the ACR/EULAR 2010 for early rheumatoid arthritis or ACR-1997 criteria for established RA.(Arnett et al. 1988; Aletaha et al. 2010)
- Biologic naïve RA patients who failed to achieve remission or low disease activity despite cs DMARDs monotherapy or combinations for at least 6 months.

Exclusion criteria:

- RA patients who were previously or are currently receiving biologic drugs.
- Patients with autoimmune diseases other than RA.
- Infections: tuberculosis, hepatitis B, C, other active or incompletely treated bacterial, viral or fungal infections.
- Patients with heart failure grade III-IV NYHA, interstitial pulmonary disease, untreated malignancies.

Data were prospectively gathered over a follow up period of 2 years. The following clinical data were recorded; patients’ demographics, simplified 28 swollen tender joint score Disease Activity Score (DAS 28), ACR 50 response, disability score using health assessment questionnaire (HAQ score of 0–3 based on severity of functional impairment), visual analogue scale pain score (VAS-pain with 0 = no pain and 10 = very severe pain), assessment for the presence of radiographic erosions and serology profile (rheumatoid factor, anti-citrullinated C peptide) were recorded. The primary outcome measure of the study was to identify responders who achieved the European League Against Rheumatism (EULAR) good responses as assessed by DAS score at 6 months (VAN GESTEL AM et al. 1996) and survey the major differences between responders versus non responders that favored an early good response or remission, ACR-50 responses at 6 months were additionally taken. The secondary outcome measure was to identify the percentage of patients sustaining their response over 2 years. Patients who fail to respond to the introduced 1st TNF-I or lose response to one anti TNF will be classified as non- responders.

### Statistical analysis

Statistical analysis was performed using SPSS (Statistical Package for the Social Science; SPSS Inc., Chicago, IL, USA) version 19 for Microsoft Windows. Quantitative data were statistically described in terms of mean and standard deviations (±SD), qualitative data were presented as median and interquartile ranges. Within group comparison of quantitative variables was done using Anova F-test with posthoc multiple group comparisons. For comparing categorical variables, Chi square (*χ*^2^) test was performed. Exact test was used instead when the expected frequency is less than 5. P values less than 0.05 was considered statistically significant. Pearson correlation was used for quantitative variables, spearman rank correlation was used for qualitative variables. Multivariate logistic regression analysis was used for identification of factors associated with good EULAR response/ remission response at 6 months and predictors of sustained response at 2 years in the study group.

## Results

A total of 80 patients were included in the study 64 females (80%) and 16 males (20%), having a mean age of 48.4 + −17.91 years a mean disease duration of 7.29 + −3.93 years and a mean baseline DAS-28 of 6 ± 1, HAQ of 1.25 + −0.35, mean ESR of 51.00 ± 25.30 mm/hr, mean CRP of 35.18 + −31.65 mg/l. Radiographic erosions were detected in 50% (n = 40) of the study population at baseline. The baseline demographic and clinical characteristics are displayed in Table 1.

**Table 1:** **The baseline demographic and clinical characteristics of the study group**

**Descriptive parameter**	**Range**	**Mean ± S.D**
**Age**	16-85 years	48.26 **±** 17.91
**Disease duration**	1-20 years	7.29 ± 3.93
**ESR**	8-119 mm/hr	51.00 ± 25.30
**CRP**	6-136.50 mg/l	35.18 ± 31.65
**Hemoglobin**	10-16gm/dl	11.56 ± 1.30
**Rheumatoid factor**	0-849	141.09 ± 157.88
**Anti-CCP**	0-450	230.05 ± 156.35
**DAS**	3-7.5	6.0 ± 1.0
**HAQ**	0.5-8	1.30 ± 0.71
**VAS**	5-10	6.70 ± 0.95
**Medications:**	Number of patients	Percentage%
**Conventional DMARDs**	58	72.5
**Methotrexate**	64	80
**Oral corticosteroids**	53	66.2
**Non-steroidal drugs**	80	100

*Abbreviations:* *ESR:* erythrocyte sedimentation rate, *CRP:* C- reactive protein, *Anti-CCP:* anti-citrullinated C peptide, *DAS-28:* disease activity score, *HAQ:* health assessment questionnaire, *VAS:* visual analogue scale for pain.

### At 6 months from initiation of therapy results showed that

Thirty patients were receiving adalimumab, 30 patients receiving infliximab and 20 patients receiving etanercept. Results of the study revealed that by completion of 6 months from initiation of therapy 70% of the patients (56) achieved the EULAR good response, with 51.8% patients (n = 29) of the 56 responders having achieved DAS-28 definition of remission (<2.6). The baseline differences between the EULAR good responders and the inadequate responders are displayed in Tables 2 and 3.

**Table 2:** **The differences between responders and non responders at inclusion in the study (0 months of follow up)**

**Parameter**	**Responders (n = 56)**	**Non-responders (n = 24)**	**Significance**
	**Mean ± S.D**	**Mean ± S.D**	**P-value**
**Age**	47.84 ± 18.89	49.64 ± 15.70	P > 0.05
**Disease duration**	7.66 ± 4.11	6.59 ± 3.41	P > 0.05
**Rheumatoid factor titers**	169.95 ± 176.19	73.80 ± 74.58	P < 0.01^**^
**ESR**	51.40 ± 24.70	49.97 ± 26.93	P > 0.05
**C-reactive protein**	36.12 ± 34.27	33.10 ± 25.19	P > 0.05
**Baseline-HAQ**	1.25 ± 0.33	1.24 ± 0.34	P > 0.05
**Baseline DAS-28**	6.32 ± 0.62	5.34 ± 1.38	P < 0.01^**^
**Baseline VAS**	6.77 ± 0.97	6.51 ± 0.90	P > 0.05
**Hemoglobin**	11.59 ± 1.30	11.36 ± 1.06	P > 0.05

*P-value: significant < 0.05. **P-value: highly significant < 0.01. *Abbreviations:* *ESR:* erythrocyte sedimentation rate, *HAQ:* health assessment questionnaire, *DAS:* disease activity score, *VAS:* visual analogue scale.

**Table 3:** **The main differences in the defined assessment measures with TNF-I at 6 months**

**Measures**	**Responders**	**Non responders**	**Significance**
			**P-value**
**ESR**	**23.56 ± 13.58**	**28 ± 16.87**	<0.05*
**CRP**	**6.66 ± 4.81**	**14.82 ± 17.90**	<0.05*
**Hb%**	**12.61 ± 1.09**	**12.01 ± 0.87**	**>0.05**
**DAS-28**	**2.80 ± 1.15**	**4.35 ± 1.24**	<0.05*
**RF at 6 months**	**118.84 ± 127.55**	**66.60 ± 74.16**	<0.05*

*P value< 0.05 is considered statistically significant.

### Analysis of correlations between the different disease related parameters and the type of response perceived with TNF-I therapy results of this study showed that

At 6 months achieving the EULAR good response using the DAS-28/ or achieving DAS-28 definition for remission directly correlated with a high positive titers of IgM rheumatoid factor and higher Hb% at baseline (r = 0.30 & 0.30, P < 0.05, respectively) and inversely correlated with baseline DAS-28 and radiographic scoring (r = −0.81 & -0.75, P < 0.01 respectively).

### At 2 years

Results showed that 38 patients out of the initially responding 56 patients (corresponding to 47.5% of the population studied, 67.8% of the responders) were still sustaining their initial good response by the completion of 2 years of follow up. Eighteen patients were considered as non-responders including 10 patients who developed secondary loss of response and were shifted to a second line biologic, while 8 patients dropped out of the study. Results showed that a sustained response at 2 years positively correlated with sero-positivity to RF and baseline Hb% (r = 0.30 & 0.33, P <0.05 respectively) and inversely correlated with baseline DAS-28, HAQ and radiographic erosions (r = −0.85, −0.74, −0.77 respectively, P < 0.01). The details of the correlations between the different assessment parameters and the treatment outcome in the studied patients are displayed in Table 4.

**Table 4:** **The correlations between the different assessment parameters and the treatment outcome in the studied patients**

**Parameter**	**EULAR good response**	**Remission**	**Sustained response**
	**At 6 months**	**At 6 months**	**At 2 years**
**Disease duration**	r = − 0.16	r = −0.05	r = − 0.10
	P > 0.05	P > 0.05	P > 0.05
**Positive IgM RF at baseline**	r = 0.30	r = 0.31	r = 0.30
	P = < 0.05^*^	P < 0.05^*^	P < 0.05^*^
**Positive Anti-CCP at baseline**	r = 0.18	r = 0.08	r = 0.14
	P > 0.05	P = 0.35	P > 0.05
**Baseline Hemoglobin**	r = 0.39	r = 0.30	r = 0.33
	P < 0.05*	P < 0.05^*^	P < 0.05^*^
**Baseline DAS 28**	r = − 0.81	r = − 0.74	r = − 0.85
	P < 0.01^**^	P <0.01^**^	P < 0.01^*^
**Baseline HAQ**	r = − 0.08	r = − 0.18	r = − 0.74
	P > 0.05	P < 0.05^*^	P < 0.01^**^
**Baseline VAS**	r = 0.12	r = − 0.06	r = − 0.17
	P > 0.05	P > 0.05	P > 0.05
**Baseline Radiographic erosions**	r = − 0.75	r = − 0.80	r = − 0.77
	P < 0.01^**^	P = 0.01^**^	P <0.01^**^
**Declining RF titers**	r = 0.33	r = 0.30	r = 0.30
	P <0.05*	P < 0.05*	P < 0.05*

*P-value: significant < 0.05. **P-value: highly significant < 0.01. *Abbreviations:* *ESR:* erythrocyte sedimentation rate, *HAQ:* health assessment questionnaire, *DAS:* disease activity score, *VAS:* visual analogue scale.

### Multivariate regression analysis

After adjustment for confounders showed that the concomitant use of methotrexate was identified as a positive predictor of good EULAR response and DAS-28 remission at 6 months (ORs = 1.13, 1.30, CI = 0.15-2.2, 0.37-10.8, respectively), also the use of csDMARDs combinations was associated by EULAR good response (ORs = 1.35, CI = 0.07-7.36). On the other hand, a higher baseline DAS was identified as a negative predictor for a good EULAR (ORs = 1.20, CI = 3.23-31.78). However, the use of oral corticosteroids didn’t prove to affect the type of response in the studied patients (P > 0.05). The study couldn’t find any significant predictive value for variations in age, gender, or disease duration.

At the end of the study period (completion of 2 years follow up) results of regression analysis showed that the presence of higher baseline serum hemoglobin concentrations, sero-positivity to IgM rheumatoid factor were in favor of a better response yet significance value was weak whereas, a lower baseline HAQ score (corresponding to lesser functional disability) and the absence of radiographic erosions at inclusion as significant predictors of sustained response to TNF-I in the studied group Table 5.

**Table 5:** **Factors related to sustained responsiveness in the study population**

**Parameter**	**Significance**
**Disease duration**	t = −1.92
	P = 0.05
**ESR**	t = −0.98
	P = 0.32
**CRP**	t = 0.09
	P = 0.92
**Hemoglobin**	t = 3.71
	P = 0.0001^**^
**Rheumatoid factor**	t = 2.17
	P = 0.03
**Anti-CCP**	t = 0.10
	P = 0.91
**DAS-28**	t = −0.27
	P = 0.78
**HAQ**	t = −2.71
	P = 0.008^**^
**VAS**	t = −2.01
	P = 0.04^*^
**Combination**	t = −1.01
	P = 0.92
**Erosive lesions**	t = −2.20
	P = 0.03^*^

*P-value: significant < 0.05. **P-value: highly significant < 0.01.

*Abbreviations:* *ESR:* erythrocyte sedimentation rate, *HAQ:* health assessment questionnaire, *DAS:* disease activity score, *VAS:* visual analogue scale.

## Discussion

The introduction and progressive developments of biologic disease modifying anti- rheumatic drugs have greatly influenced the treatment paradigm as well as treatment outcome in patients with inflammatory arthritis. Till present even with the amazing advent in this domain together with the expanding list of biologic DMARDs, anti-tumor necrosis factor therapy or tumor necrosis factor inhibitors remain on top of the list. The comparative effectiveness of the three commonly used anti-TNF infliximab, etanercept and adalimumab didn’t vary significantly as has been shown in various studies including one meta-analysis of 26 published placebo-controlled RCTs of patients with RA in MTX-resistant populations where the investigators weren’t able to show significant variations in efficacy among the 3 TNF inhibitors. (Hetland et al. 2010; Gartlehner et al. 2006) Despite the multiplicity of such published trials discussing the efficacy of biologic Tumor necrosis Factor inhibitors in refractory rheumatoid arthritis (RA), studies displaying predictors of good response to such therapy in the daily life clinical practice remain insufficient and heterogeneous demanding continued testing of such predictors within different ethnic populations and at different levels of rheumatology practice (Scott and Kingsley 2006; Taylor and Feldmann 2009; Grewal 2009; Canhão et al. 2012).

To our knowledge this study is the first to scrutinize predictors of response to TNF-I in patients with refractory RA who started their first line TNF-I and were prospectively followed up for response over 2 years in this region of KSA. This was a single center prospective observational study that included eighty RA patients who were eligible for inclusion and consented to receive TNF-I biologic therapy. The goal of this study wasn’t to measure comparative effectiveness of the three used TNF-I to each other but to investigate factors related to achieving an early EULAR good response/remission (remission is defined as DAS score < 2.6) as assessed by changes in DAS-28 score at 6 months and to pick up possible predictors of sustained response to therapy by completion of the study period (2 years). Patients who achieved a good response or went into remission at 6 months were classified as responders, whereas patients who failed to achieve a satisfying response, developed drug related serious adverse events or were shifted to another biologic were considered as non-responders. The study successfully completed the observation and recording of the desired outcome measures with results at 6 months showing that the majority of the RA population included (70%, n = 56) achieved a good EULAR response on TNF-I, with more than half of the responders 51.8% (n = 29) satisfying the DAS-28 definition of remission at 6 months. The study showed that RA patients who achieved an early response at 6 months were found to have significantly higher IgM rheumatoid factor titers at baseline compared to non-responders (mean 169.95 ± 176.19 mg/l, P <0.01). Such statistical conclusions were further supported by results of the correlations between the patterns of responses perceived during the assessment period and the baseline (at inclusion) disease related/patient related features. The study found that an early good response and/or remission at 6 month significantly correlated with higher Ig M rheumatoid factor titers and higher hemoglobin concentrations at inclusion, yet inversely correlated with the baseline DAS-28, the HAQ score and the presence of radiographic erosions. At 2 years the study showed that 67.8% of the responders were still sustaining their response to TNF-I. The pertinence of sustained response at 2 years positively correlated with sero-positivity to RF and Hb% at inclusion and inversely correlated with baseline DAS-28, HAQ and radiographic scoring. The continued use of methotrexate as an anchor drug was associated with a good EULAR response and DAS-28 remission at 6 months, also the use of conventional synthetic DMARDs (cs DMARDs) in combination with the used TNF-I was associated by EULAR good response and ACR 50 responses. However, the use of oral corticosteroids (prednisolone) wasn’t shown to affect the type of response in the studied patients. Higher baseline DAS was identified as a negative predictor for a good EULAR and DAS remission, sustained response and/or ACR 50 responses. Disease duration didn’t appear to affect the response to biologic therapy in the studied population. The study couldn’t find any significant predictive value for variations in age, gender, or disease duration among the studied RA patients.

Results of the current study didn’t seem far from that reported in late studies including some trials by the British study (BSRBR) by Hyrich et al. 2006, GISEA study by Mancarella et al. 2007, the ReAct study by Bombardieri et al. 2007, Kristensen et al. 2008 and the German study by Kleinert et al. 2012, *amongst others* in several points particularly regarding continued use of baseline methotrexate or conventional DMARDS in combination with biologics. (Kristensen et al. 2008; Hyrich et al. 2006; Kleinert et al. 2012; Mancarella et al. 2007; Bombardieri et al. 2007; Breedveld et al. 2006; Maini et al. 1999; Van der Heijde et al. 2007; St Clair et al. 2004; Nozaki et al. 2010; Potter et al. 2009) The authors could identify the use of methotrexate and conventional DMARDs as significant contributors to good clinical outcomes a conclusion shared by the current study. Additionally important results of the current study illustrated that a low HAQ corresponding to a better functional status and low DAS score at inclusion favored a good early response to biologic TNF-I. Unlike that reported by Hetland et al. 2010, were the authors found that older age and concomitant use of corticosteroids were negative predictors of good response, (Hetland et al. 2010) the study wasn’t able to detect any significant influence of age, gender or oral corticosteroid use on the pattern or timing of responses recorded.

The existence of RF especially IgM-RF (Abs to conserved region of IgG class Igs) with the combined detection of additional isotypes IgA-RF remain one of the diagnostic criteria in RA and in multiple reports its’ existence in high titers corresponded to severe erosive disease mandating aggressive therapy. There has been a lot of conflicting results regarding the influence of TNF-I and conventional DMARDs on serum RF levels in RA as well as the possible influence of higher titers of RF on the pattern of responses to TNF-I. The exact mechanisms by which TNF-I affect rheumatoid factor remains unclear as it is not known whether TNF-I are capable of direct blockage of RF production or not. However, infliximab therapy has been proven to reduce the number of synovial infiltration cells, including plasma cells. RF-producing cells are present in the inflamed rheumatoid synovium and because the local environment may favour synovial RF production, rheumatologists might expect that the reduction in inflammatory lymphoplasmacytic infiltration into the rheumatoid synovium would contribute to a reduced production of RF. A number of published studies coincided in the finding that a positive IgM RF was related to a poor response to TNF-I, other studies found a correlation between the declining levels of autoantibodies with TNF-I and the response, whilst in a late study by Klaasen et al. 2009 and another meta-analysis by Salgado et al. 2014 the investigators reported that a positive RF status doesn’t correlate with the EULAR good response or remission. In the current study the investigators identified that patients with higher baseline IgM rheumatoid factor and higher baseline hemoglobin achieved a better response on anti-TNF therapy, however the correlations were of moderate to weak significance furthermore, the achievement of good response and remission correlated with the decline in rheumatoid factor titers which was also a weak correlation. Such finding has been reported in some other studies (Nozaki et al. 2010; Potter et al. 2009; Klaasen et al. 2009; Salgado et al. 2014; Atzeni et al. 2006; Yazdani-Biuki et al. 2005; Smeets et al. 2003; Klaasen et al. 2011; Bruns et al. 2009; Bobbio-Pallavicini et al. 2007).

Because results of clinical trials do not always mirror an exact of what rheumatologists face in real life daily practice, observational studies and short reports still represent an important contributor to the knowledge bank in the field of research concerning the use of biologic DMARDs in refractory RA. Despite that researchers might face the challenge that such studies are usually open labelled non-randomized which might generate methodological limitation such drawback can be minimized by avoidance of separate analysis.

## Conclusions

In this study, a lower baseline DAS-28 and HAQ scores as well as the absence of radiographic erosions showed a highly significant correlation with good EULAR response, remission and sustained responses. While a higher baseline hemoglobin concentrations, a lower baseline HAQ score and the absence of radiographic erosions were identified as significant predictors of sustained responses to TNF-I in the studied patients with refractory RA. The use of combination therapy particularly with methotrexate favored an earlier good response and remission by 6 months. Good response to TNF-I and sustained responses showed some correlation with the decline in RF titers during observation a finding that demands further investigations in upcoming researches.

The limited sample size represents one limitation of the study and authors recommend encouraging similar studies in the field to provide a sufficient bank of evidences regarding predictors of response to first and second line biologics in refractory disease.
